# Combined Effects of Elevated O_3_ Concentrations and Enhanced UV-B Radiation of the Biometric and Biochemical Properties of Soybean Roots

**DOI:** 10.3389/fpls.2017.01568

**Published:** 2017-09-11

**Authors:** Bing Mao, Yan Wang, Tian-Hong Zhao, Rong-Rong Tian, Wei Wang, Jia-Shu Ye

**Affiliations:** ^1^Postdoctoral Research Station of Crop Science, College of Agronomy, Shenyang Agricultural University Shenyang, China; ^2^College of Agronomy, Shenyang Agricultural University Shenyang, China; ^3^National Field Observation and Research Station of Shenyang Agro-Ecosystems Shenyang, China

**Keywords:** flavonoids, endogenous hormones, enzyme activities, soybean roots, open-top chambers

## Abstract

Enhanced ultraviolet-B (UV-B) radiation and elevated tropospheric ozone alone may inhibit the growth of agricultural crops. However, research regarding their combined effects on growth and biochemical properties of roots is still scarce. Using open top chambers, we monitored the response of growth, secondary metabolites, endogenous hormones and enzyme activities of soybean roots to elevated O_3_ and enhanced UV-B individually and in combination at stages of branching, flowering and podding. Our results indicated that the root biomass decreased by 23.6, 25.2, and 27.7%, and root oxidative capacity declined by11.2, 39.9, and 55.7% exposed to elevated O_3_, enhanced UV-B, and O_3_ + UV-B, respectively, compared to the control treatment. Concentrations of quercetin and ABA were significantly increased, while concentrations of total polyphenol and P-coumaric acid responded insignificantly to elevated O_3_, enhanced UV-B, and O_3_ + UV-B during the whole period of soybean growth. Elevated O_3_, enhanced UV-B and O_3_ + UV-B showed significant negative effects on superoxide dismutase (EC 1.15.1.1) activity at flowering stage, on activities of peroxidase (EC 1.11.1.7) and catalase (EC 1.11.1.6) at podding stage, on ascorbate peroxidase activity during the whole period of soybean growth. Moreover, compared to hormones and enzyme activity, secondary metabolisms showed stronger correlation with root growth exposed to elevated O_3_ and enhanced UV-B individually and in combination. Our study concluded that combined effects of O_3_ and UV-B radiation significantly exacerbated the decline of soybean root growth, and for annual legumes, the inhibited root growth exposed to O_3_ and/or UV-B radiation was mostly associated with secondary metabolisms (especially flavonoids).

## Introduction

Tropospheric O_3_ has increased worldwide as a result of industrialization and anthropogenic activities, and its concentration has already exceeded the threshold levels for the protection of vegetation (Vingarzan, 2004). The increase of tropospheric O_3_ concentration can reduce plant growth and crop yields not only by its direct injury on leaves and alteration of photosynthetic systems, but also indirectly through its impacts on resource allocation (Goumenaki et al., 2010). Moreover, global changes in the chemical composition of the atmosphere with a substantial reduction of the stratospheric O_3_ layer have led to a notable increase in the solar UV-B reaching the earth surface (Ballaré et al., 2011). Although stratospheric O_3_ has been slowly recovering, no statistically significant decreases in UV-B radiation attributable to the recovery of stratospheric O_3_ have yet been detected (Bais et al., 2015). Numerous studies have shown that enhanced UV-B radiation can affect physiological and biochemical processes of many plant species (Jansen et al., 1998; Newsham and Robinson, 2009; Bussell et al., 2012; Li X. et al., 2012; Mazza et al., 2013). Under natural field conditions, plants are usually affected by elevated O_3_ and enhanced UV-B radiation simultaneously. However, most of our knowledge today depends on the individual effects of O_3_ and UV-B radiation on the productivity and quality of some important agricultural crops and plants, and studies regarding their combined effect on crop yields and plant growth are still scarce (Ambasht and Agrawal, 2003; Tripathi et al., 2011; Rinnan et al., 2013; Tripathi and Agrawal, 2013).

Several studies regarding combined effect of enhanced UV-B and elevated O_3_ on physiological characters and yield components in different plant species have found a wide variety of responses. For example, Miller et al. (1994) did not find any effect of enhanced UV-B (3.02, 6.24 and 8.98 kJ m^-2^) on soybean yield in the presence or absence of O_3_ (24, 49 and 83 nL L^-1^). Ambasht and Agrawal (2003) noticed negative effects of combination of elevated O_3_ concentration (0.07 μmol mol^-1^) and enhanced UV-B radiation (7.1 kJ m^-2^) on biomass, yield, photosynthetic rate, chlorophyll, carotenoid and ascorbic acid contents and CAT activity, whereas positive effects on total phenol content and POX activity. However, most of these studies focused on the above-ground system. Compared to above-ground responses, below-ground responses to O_3_ may be larger in magnitude and may have larger, long-term consequences for ecosystem productivity and carbon, nutrient and water cycling (Andersen, 2003; Laurence and Andersen, 2003). Similarly, previous study showed that the growth of root was also affected by enhanced UV-B radiation (Xu et al., 2013; Yokawa et al., 2013; Yokawa and Baluška, 2015). However, little is known about the root responses to the combined effects of elevated ozone and enhanced UV-B radiation.

It is well known that plants generate antioxidant enzymes (Mittler, 2002; Blokhina et al., 2003), such as SOD, APX, POD, glutathione reductase (GR), guaiacol peroxidase (GPX), PAL, and CAT, and protective secondary metabolites (Paul and Gwynn-Jones, 2003; Mewis et al., 2012; Escobar-Bravo et al., 2017; Wang et al., 2017), such as flavonoids and phenolic acids, in response to environmental changes. Phenolic acids (such as ferulic acid and P-coumaric acid) are mainly responsible for providing plant pest and disease resistance and protection from UV radiation. Flavonoids (such as quercetin and rutin) in root exudates are inducers of genes responsible for the nodulation process of rhizobium in specific legumes and are responsible for triggering action and signaling cascades (Walker et al., 2003; Peer et al., 2004). Previous studies have showed that UV-B exposure might initiate signaling through UV RESISTANCE LOCUS 8 (UVR8) and then induce the changes of metabolites and antioxidant enzymes involved in protection against enhanced UV-B radiation (Binder et al., 2009; Hao et al., 2009; Huang et al., 2016; Liu et al., 2016). Several studies suggest that elevated O_3_ concentration induced oxidative damage in plant cells leading to changes in concentrations of secondary metabolites and activities of enzymes (Rai and Agrawal, 2008; Tripathi et al., 2011). Furthermore, plant hormones play important roles in regulating developmental processes and signaling networks involved in plant responses to environmental change. When plants are subject to environmental change, the concentrations of some plant endogenous hormones might be different, and the differences of endogenous hormones not only influence the adaptive response to environmental changes but also affect the normal growth and development (Dodd, 2005; Albacete et al., 2008; Li et al., 2011). Unfortunately, the changes of concentrations of secondary metabolites, hormones and activities of enzymes during the growth of roots of plants under coupling condition of elevated O_3_ concentration and enhanced UV-B radiation remain largely unknown.

Soybean (*Glycine max*) ranks among the most O_3_-sensitive agricultural crops (Mills et al., 2007). The effects of enhanced O_3_ concentrations and enhanced UV-B radiation on aboveground growth, morphology and yield of soybean have been studied widely (Li et al., 2002; Kakani et al., 2003; Feng and Kobayashi, 2009; VanLoocke et al., 2012; Kataria et al., 2017). For instance, Ashmore (2005) found that an increase of O_3_ concentration from 30 to 60 ppb for 7 h day^-1^ could decrease soybean yield by 16%. Liu et al. (2013) found that enhanced UV-B radiation (13 kJ m^-2^ d^-1^) decreased plant height, dry weight of individual stem and yield per plant of three soybean cultivars on average by 15.5, 16.9, and 43.7%, respectively. However, the effects of elevated O_3_ concentrations and/or enhanced UV-B radiation on root growth of soybean have not been investigated. Furthermore, a soybean developmental stage classification based on significant changes in external morphological characteristics has been in place since 1971 and plays important roles in soybean cultivation (Fehr et al., 1971). The root growth at different developmental stages (such as branching stage, flowering stage, and podding stage) is critical in agriculture and ecology, while little is known about the effects of elevated O_3_ concentrations and/or enhanced UV-B radiation on root growth of soybean at different developmental stages.

The objective of this study was to examine how elevated O_3_ concentrations (110 ± 10 nmol mol^-1^ for 8 h per day) and/or enhanced UV-B radiation (9.50 kJ d^-1^ m^-2^ for 8 h per day) affect secondary metabolites, endogenous hormones and enzyme activities of soybean roots at stages of branching, flowering and podding (from June 20 to August 12), using the open top chambers (OTC). We hypothesized that: (1) elevated O_3_ and enhanced UV-B individually or in combination inhibited root growth via changes of secondary metabolites, endogenous hormone and enzyme activities, and (2) the effect of O_3_ + UV-B is synergistic on root growth.

## Materials and Methods

### Experimental Site and Design

The experimental site is located in the Shenyang Experimental Station of Ecology, Chinese Academy of Sciences (41°310′N, 123°220′E). This region has a continental monsoon climate with a mean annual temperature of 7.0–8.0°C, annual precipitation of 650–700 mm, and an annual non-frost period of 147–164 days. The soil at the study site is classified as an aquic brown soil (silty loam Hapli-Udic Cambosols in Chinese Soil Taxonomy), with 11.28 g kg^-1^ organic C, 1.20 g kg^-1^ total N, 0.41 g kg^-1^ total P, pH (H_2_O) 6.7 at 0–15 cm depth.

### Experimental Design

The study was conducted on soybean (*G. max*) plants grown in open-top chambers (OTCs), which were established in 2008. The OTCs were 1.15 m in diameter and 2.4 m in height with a 45° sloping frustum and the minimum distance between any two chambers was 4 m. Each chamber was constructed from an iron framework, clad with standard horticultural glass, with a plenum incorporated just below the mouth of the chamber (at 2.4 m from ground-level) to reduce incursion of ambient air. From June to September, Mean temperature of the day was about 25.6°C and mean relative air humidity throughout the day was 50.6% in the OTCs. The mean value of UV-B radiation was about 8.64 kJ d^-1^ m^-2^ during clear sky conditions from June to September in the OTCs. The mean value of O_3_ concentration was about 45 ± 5 nmol mol^-1^ and the average value of *AOT*40 was 3.5 ppm h during clear sky conditions from June to September in the OTCs.

The experimental design was based on completely randomized plots including four treatments belonging to 12 OTCs: (1) control (hereinafter referred to as CK, O_3_ concentration, 45 ± 5 nmol mol^-1^ + UV-B radiation, 8.64 kJ d^-1^ m^-2^); (2) elevated O_3_ concentration (110 ± 10 nmol mol^-1^ + UV-B radiation, 8.64 kJ d^-1^ m^-2^); (3) enhanced UV-B radiation (9.50 kJ d^-1^ m^-2^ + O_3_ concentration, 45 ± 5 nmol mol^-1^); (4) O_3_ + UV-B (elevated O_3_ concentration, 110 ± 10 nmol mol^-1^ + enhanced UV-B radiation, 9.50 kJ d^-1^ m^-2^). There were no air filtered chambers in all OTCs. Each treatment had three replicated OTCs and each OTC was divided into three compartments in order to avoid variability within the same chamber, so there were three replications for each treatment.

For the elevated O_3_ treatment and the combined treatment, O_3_ was generated from pure oxygen by an electrical discharge O_3_ generator (GP-5J, China) and then mixed with ambient air in the OTCs to achieve the target O_3_ concentration. The O_3_ concentrations were continuously monitored by O_3_ analyzers (S-900 Aeroqual, New Zealand), and were controlled by computers using a professional program for O_3_ dispensing and monitoring (Zhao et al., 2010). Meanwhile, during O_3_ fumigation from June to September in the OTCs, the mean of the daily 8 h O_3_ concentration (M8) in elevated O_3_ treatment averaged 114.4 nmol mol^-1^ with a maximum of 125.1 nmol mol^-1^ and in combined treatment averaged 115.7 nmol mol^-1^ with a maximum of 124.0 nmol mol^-1^. In addition, the average value of *AOT*40 in elevated O_3_ treatment and combined treatment during the whole fumigation season was 37.3 and 37.7 ppm h, respectively.

**FIGURE 1:**
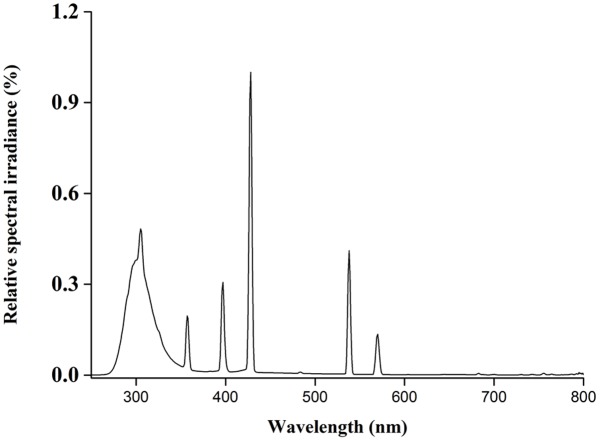
The relative spectral irradiance of the UV-B lamps.

UV-B was artificially supplied with UV-B radiation by 40 W fluorescent tubes (F40T8/30; length was 120 cm; Beijing Lighting Research Institute, peak value was 305 nm; **Figure 1**) held in mobile and adjustable frames over each pot row. In control treatment (CK) and elevated O_3_ treatment, UV-B tubes were covered with 0.13 mm polyester filters to absorb radiation below 320 nm (UV-B + UV-C). In treatments of enhanced UV-B radiation and combined treatment, UV-B tubes were covered with 0.08 mm cellulose diacetate filters to absorb radiation below 280 nm (UV-C). The UV-B radiation of two tubes with cellulose diacetate filters on the sample surface was 9.50 kJ d^-1^ m^-2^, equivalent to those about 10% enhanced in average of UV-B radiation on the sample surface in Shenyang during clear sky conditions in summer. The polyester filters and cellulose diacetate filters were changed at week intervals. The distance between the top canopies of the plants and the lamps was maintained at 40 ± 2 cm by the mobile frames. UV radiation was monitored by UV radiometer (UV 340B, China). Plants were exposed to elevated O_3_ or/and UV-B radiations for 8 h (09:00–17:00) per day in the middle of the photoperiod from June 20 to August 12.

Soil at 0–15 cm layer was collected at the study site and then was mixed thoroughly after removing roots and organic residues. After sieving (2 mm), the soils were used in the pots of soybean cultivar. The potted soybean cultivar was Tiefeng 29, which was seeded in each pot (26 cm long × 36 cm wide × 45 cm deep) on May 20 in 2016. Five plants in three leaves stage were established in each pot. There were nine pots in total in each OTC [three collected periods (branching stage, flowering stage and podding stage) × 3 compartments]. The pots were periodically watered to prevent water deficit. Each whole pot of soil (26 cm long × 36 cm wide × 45 cm deep), which contained the whole roots of five plants, was collected at branching stage (June 30, 2016), flowering stage (July 24, 2016), and podding stage (August 12, 2016). Each whole pot of soil was immersed in water for about 60 min and then roots and root nodules were carefully collected in a 0.5 mm sieve with the aid of running water. Debris, weeds, and dead roots were removed by hand. The samples of root and root nodules were then combined so that there was one root sample for each pot. The roots and root nodules were blotted dry with tissue paper and biomass of roots and root nodules was measured. Then each root sample was divided into two sub-samples. One (fresh sample) was stored at 4°C to analyze root activity and the relative electrical conductivity and the other (frozen sample) was immediately frozen in liquid nitrogen and stored at -70°C until further analysis.

### Biochemical Analyses

Lipid peroxidation in soybean root was determined by measuring MDA formation, using the thiobarbituric acid (TBA) method as previously described by Buege and Aust (1978). Frozen roots (500 mg) were ground into a fine powder and then were homogenized in trichloroacetic acid (TCA). After centrifugation, the supernatants were mixed with 0.5% TBA (prepared in 20% TCA). The mixture was incubated at 95°C for 30 min and the reaction was stopped by placing the mixture on ice for 5 min. After centrifugation, the absorbance of the supernatant was measured at 532 and 600 nm. After subtracting the non-specific absorbance (*A*_600 nm_), the MDA concentration was determined by its extinction coefficient of 155 mM^-1^ cm^-1^ and expressed as nmol g^-1^ of fresh weight.

Fresh roots (200 mg) were washed with no ion water and were cut into tubes with 20 mL of ionized water. After shaking 30 min, DDS-11A Type conductivity meter was used to determine the conductivity as *E*_1_. And then the solutions were incubated at boiling water bath for 10 min. After cooling, the solutions were used to determine the total conductivity as *E*_2_. No ion water conductivity was considered as *E*_0_. The relative electrical conductivity (*R*) is calculated according to the method of Zhao et al. (1992) as: *R* = [(*E*_1_ - *E*_0_)/(*E*_2_ - *E*_0_)] × 100%.

Root activity was analyzed by a modified α-naphthylamine oxidation method (Sakai and Yoshida, 1957; Ando et al., 1983). Fresh roots (1 g) were immersed in 50 μg mL^-1^ of α-naphthylamine solution for 2 h at 25°C. 2 mL of α-naphthylamine solution was pipetted out and reacted with 10 mL of 0.1% sulfanilic acid and 2 mL of NaNO_2_. The absorbance of the colored solution was determined at 530 nm.

Total flavonoids concentration was determined by modified method of Geissman (1955). Frozen samples (500 mg) were extracted with ethanol for 8 h and then the sample solution was distilled. After washed by ether, the sample solution was measured by spectrophotometry at 510 nm. Total polyphenol concentration was determined by the Folin-Ciocalteu method (Waterman and Mole, 1994). In brief, frozen sample (500 mg) with folin reagent and Na_2_CO_3_ was incubated at 75°C in a water bath for 10 min after extracted by 60% methanol for 2 h. Then the sample was measured by spectrophotometry at 760 nm. An Agilent (Waldbronn, Germany) 1100 HPLC series, which consists of a degasser, binary pump, auto-sampler, thermostat, and photodiode array detector, with a C18-column (Hypersil ODS, 250 mm × 4.6 mm) was used to determine the concentration of rutin, quercetin, ferulic acid and P-coumaric acid (Montedoro et al., 1992; Huang et al., 2016). Pure compound of rutin, quercetin dehydrate, ferulic acid and P-coumaric acid (Sigma, China) were used as external standards.

Extraction, purification, and determination of endogenous levels of IAA, ABA, and ZR by an indirect ELISA technique were performed as described by Teng et al. (2006). The frozen samples (1 g) were ground under liquid nitrogen, extracted with ice-cold 80% methanol (v/v) containing 1 mmol L^-1^ butylated hydroxytoluene to prevent oxidation, and then stored overnight at 4°C for 16 h in the dark. After centrifugation at 4°C, the supernatants were passed through a C18 Sep-Pak cartridge (Waters, Milford, MA, United States). The efflux was collected and dried in N_2_, and dissolved in 0.01 mol L^-1^ phosphate buffer solution (pH 7.4) and concentrations of IAA, ZR, and ABA were determined in an enzyme-linked immunosorbent assay (ELISA) following methods described in previous publications (Yang et al., 2001; Zhu et al., 2005).

Activities of SOD (EC 1.15.1.1) were assessed by the method of Beauchamp and Fridovich (1971). The frozen samples (500 mg) were immersed in EDTA–phosphate buffer (pH 7.8). After filter and centrifugation, the supernatant was used to determine SOD activity by inhibition of the photochemical reduction of nitro-blue tetrazolium (NBT) at 560 nm. POD (EC 1.11.1.7) was extracted by homogenizing 500 mg frozen roots with 5 ml 0.1 mol L^-1^ Tris-HCl buffer (pH 8.5) at 0°C. After filter and centrifugation, the supernatant with 0.2 mol L^-1^ phosphate buffer (pH 6.0), 0.028 mL H_2_O_2_ and 0.019 mL guaiacol was used to determine POD activity at 470 nm (Kochba et al., 1977). APX activity was determined by the method of Nakano and Asada (1981). 500 mg frozen roots were homogenized in 50 mmol L^-1^ phosphate buffer (pH 7.6), 0.1 mmol L^-1^ EDTA, 0.5 mmol L^-1^ ascorbate and 0.1 mmol L^-1^ H_2_O_2_. After centrifugation, the supernatant was used to determine APX activity at 290 nm.

Catalase (CAT; EC 1.11.1.6) activity was extracted by homogenizing 500 mg frozen roots at 4°C in 100 mol L^-1^ cold phosphate buffer (pH 7.2) containing 0.5% Triton-X. Activity of CAT was assessed in 100 μL phosphate buffer, 400 μL 200 mmol L^-1^ H_2_O_2_ and 100 μL enzyme extract. A decrease in H_2_O_2_ was measured at 240 nm, and activity of CAT was measured according to the method of Aebi (1984). Phenylalanine ammonia-lyase (PAL, EC 4.3.1.5) activity was determined by the method of Havir and Hanson (1968) and Bradford (1976). The calculation was based on the extinction coefficient (9500 M^-1^ cm^-1^) for *trans*-cinnamic acid. One unit of activity for PAL was defined as the amount of enzyme which caused the formation of 1 μmol *trans*-cinnamic acid per hour. Lipoxygenase (LOX) was determined by the method of Buranasompob et al. (2007). 500 mg frozen roots were used for the extraction of LOX in phosphate buffer (pH 7.5). After added solid ammonium sulfate and centrifugation, the supernatant was used to determine APX activity.

### Statistical Analysis

The differences of MDA concentration, relative electrical conductivity, root biomass, root nodule biomass and root activity in the four treatments of CK, elevated O_3_, enhanced UV-B radiation and O_3_ + UV-B were evaluated by one-way analysis of variance (ANOVA) (SPSS 16.0). Multiple comparisons among means of MDA concentration, relative electrical conductivity, root biomass, root nodule biomass and root activity under the four treatments were performed with the Tukey’s multiple comparisons test. Three-way ANOVA was used to evaluate the effects of the elevated O_3_ concentration, enhanced UV-B radiation and growth stage (branching, flowering and podding stages) on root growth, MDA concentration, relative electrical conductivity, secondary metabolisms, endogenous hormones, and enzyme activities. Biometric and biochemical properties of soybean roots not meeting assumptions of normality and homogeneity of variance were log-transformed before statistical analysis. We used paired T test to analyze the differences between control treatment and the treatments of elevated O_3_, enhanced UV-B and O_3_ + UV-B in secondary metabolisms, endogenous hormones and enzyme activities of soybean roots at stage of branching, flowering and podding. Multivariate analyses were analyzed using Canoco 5.0. Six secondary metabolisms, six enzymes and six hormones of soybean at four treatments were log-transformed (using the “standardize species” option in Canoco 5.0) before unconstrained principal component analysis (PCA). Furthermore, to better highlight the effects of biochemical properties (secondary metabolisms, endogenous hormones, and enzyme activities) of soybean roots on root growth (root biomass and root nodule biomass) and root activities, constrained redundancy analyses (RDA) were used. Significance was based on permutation test using 999 permutations and using a split-plot design (Lepš and Šmilauer, 2003; Li et al., 2015).

## Results

### Root Growth and Activity

**FIGURE 2:**
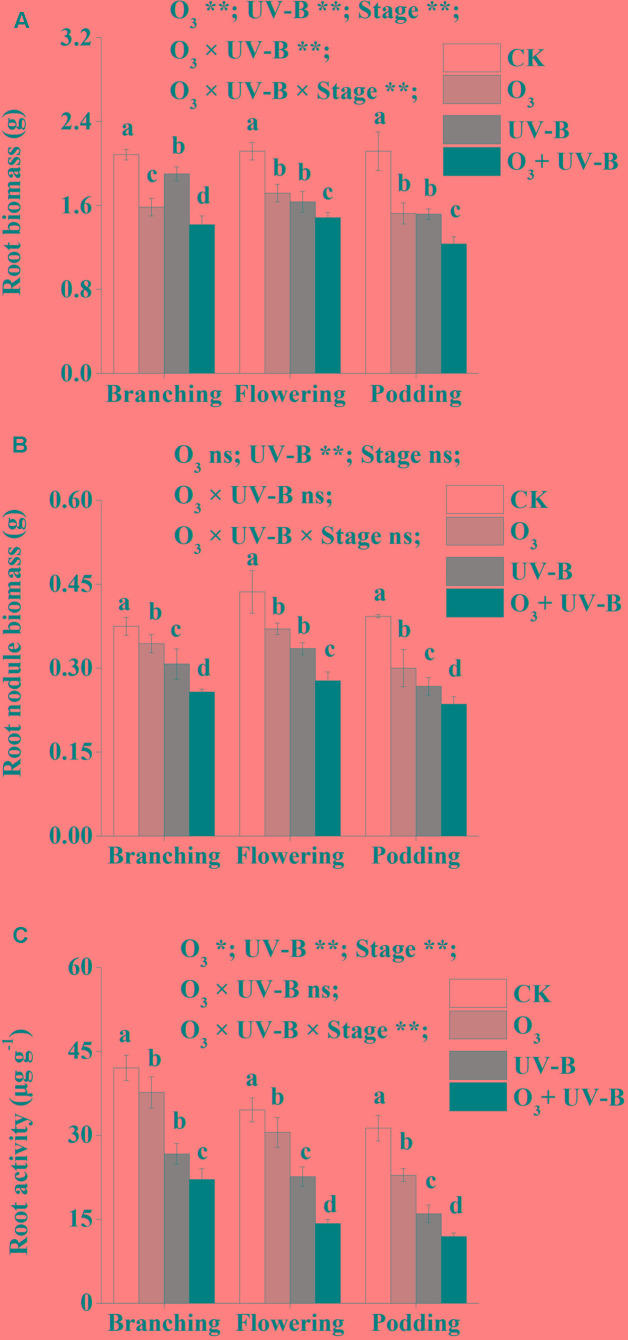
Root biomass **(A)**, root nodule biomass **(B)** and root activity **(C)** of soybean in response to control treatment (CK), elevated O_3_ treatment (O_3_), enhanced UV-B radiation treatment (UV-B) and O_3_ + UV-B treatment (O_3_ + UV-B) at stages of branching, flowering and podding. Different letters above the bars represent significant differences from Tukey’s multiple comparisons among four treatments (*P* < 0.05). For each parameter, results of three-way ANOVA are reported, with asterisks showing the significance of factors [elevated O_3_ treatment (O_3_), enhanced UV-B treatment (UV-B) and growth stage of soybean (Stage)] and their interaction for: ^∗∗^*P* < 0.001, ^∗^*P* < 0.05, ns: insignificant.

Biomass of root and nodule as well as root activity were significantly lower in the treatments of elevated O_3_, enhanced UV-B radiation and O_3_ + UV-B than CK treatments during the whole period of soybean growth (**Figure 2**). There were no significant differences in root biomass between elevated O_3_ treatment and enhanced UV-B treatment at stages of flowering and podding (**Figure 2A**). Root biomass, nodule biomass and root activity were significantly lower in O_3_ + UV-B treatment than those in the other three treatments during the whole period of soybean growth (**Figure 2**).

Furthermore, the interaction of elevated O_3_ concentration and enhanced UV-B radiation was found to be significant for root biomass (O_3_ × UV-B term at *P* < 0.001; **Figure 2**). The interactive effects was found to be insignificant for the parameters of root nodule biomass and root activity (O_3_ × UV-B term at *P* = 0.074, *P* = 0.492, respectively).

### MDA and Relative Electrical Conductivity of Soybean Roots

**FIGURE 3:**
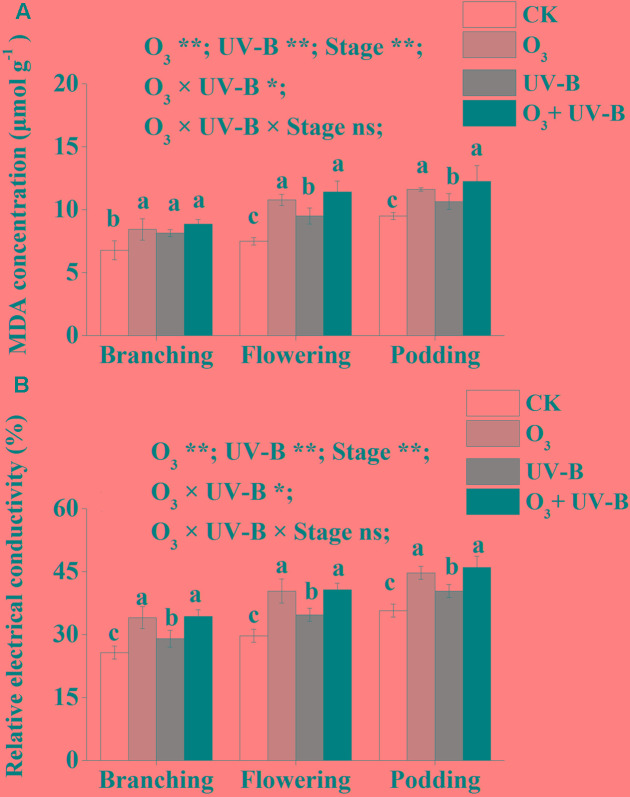
Malondialdehyde concentration (μmol g^-1^) **(A)** and relative electrical conductivity (%) **(B)** of soybean roots in response to control treatment (CK), elevated O_3_ treatment (O_3_), enhanced UV-B and O_3_ + UV-B treatment (O_3_ + UV-B) at stages of branching, flowering and podding. Different letters above the bars represent significant differences from Tukey’s multiple comparisons among four treatments (*P* < 0.05). For each parameter, results of three-way ANOVA are reported, with asterisks showing the significance of factors (elevated O_3_ treatment (O_3_), enhanced UV-B and growth stage of soybean (Stage)] and their interaction for: ^∗∗^*P* < 0.001, ^∗^*P* < 0.05; ns, insignificant.

Compared to CK treatment, elevated O_3_, enhanced UV-B and elevated O_3_ + enhanced UV-B increased MDA concentration and relative electrical conductivity of soybean roots during the whole period of soybean growth (**Figure 3**). There was no significant difference in MDA concentration and relative electrical conductivity exposed to elevated O_3_ and elevated O_3_ + enhanced UV-B during the whole period of soybean growth. Furthermore, the interaction of elevated O_3_ and enhanced UV-B was found to be significant for MDA concentration and relative electrical conductivity of soybean root (O_3_ × UV-B term all at *P* < 0.05).

### Biochemical Properties of Soybean Roots

Flavonoids concentration of soybean roots was significantly increased by exposure to O_3_ + UV-B at flowering stage and podding stage (**Table 1**). Three treatments of elevated O_3_, enhanced UV-B radiation and O_3_ + UV-B significantly increased quercetin concentration during the whole period of soybean growth. Enhanced UV-B radiation and O_3_ + UV-B showed significant positive effects on rutin concentration of soybean root at stages of flowering and podding. There were no significant differences in polyphenol and P-coumaric acid concentrations of soybean root among the four treatments. Ferulic acid concentration of soybean root was significantly increased by exposure to elevated O_3_, enhanced UV-B radiation and O_3_ + UV-B at podding stage.

**Table 1:** Differences in secondary metabolisms of soybean roots in response to control treatment (CK), elevated O_3_ treatment (O_3_), enhanced UV-B treatment (UV-B) and O_3_ + UV-B treatment (O_3_ + UV-B) at stages of branching, flowering, and podding (mean ± SD, *n* = 3).

Treatments	Branching	Flowering	Podding	Branching	Flowering	Podding
	**Total flavonoids (mg g^-1^)**			**Total polyphenol (mg g^-1^)**		
		
CK	5.31 ± 0.19	5.12 ± 0.18	5.19 ± 0.18	1.84 ± 0.07	2.29 ± 0.09	2.69 ± 0.11
O_3_	5.49 ± 0.19	5.62 ± 0.20	5.80 ± 0.20	1.87 ± 0.07	2.50 ± 0.10	2.87 ± 0.11
Percentage change	+3.77	+9.8	+11.54^∗^	+1.78	+9.18	+6.53
UV-B	5.71 ± 0.20	5.56 ± 0.20	6.10 ± 0.21	1.88 ± 0.07	2.63 ± 0.10	2.96 ± 0.12
Percentage change	+7.55	+9.8	+17.31^∗^	+1.99	+15.01	+9.9
O_3_ + UV-B	5.61 ± 0.20	5.92 ± 0.21	5.80 ± 0.21	1.83 ± 0.07	2.36 ± 0.09	2.77 ± 0.11
Percentage change	+5.66	+15.69^∗^	+11.54^∗^	-0.34	+3.35	+2.68

	**Quercetin (mg g^-1^)**			**Ferulic acid (mg g^-1^)**		
		
CK	1.13 ± 0.05	1.21 ± 0.05	1.20 ± 0.05	0.28 ± 0.01	0.34 ± 0.02	0.40 ± 0.02
O_3_	1.25 ± 0.05	1.36 ± 0.06	1.48 ± 0.06	0.28 ± 0.01	0.36 ± 0.02	0.43 ± 0.02
Percentage change	+10.62^∗^	+12.40^∗^	+23.33^∗^	+1.78	+4.19	+6.53^∗^
UV-B	1.37 ± 0.06	1.40 ± 0.06	1.50 ± 0.06	0.30 ± 0.02	0.37 ± 0.02	0.46 ± 0.02
Percentage change	+21.24^∗^	+15.37^∗^	+25.00^∗^	+8.04	+9.18	+14.80^∗^
O_3_ + UV-B	1.31 ± 0.05	1.63 ± 0.06	1.48 ± 0.06	0.28 ± 0.01	0.35 ± 0.02	0.44 ± 0.02
Percentage change	+15.58^∗^	+12.31^∗^	+23.33^∗^	-0.34	+3.35	+8.87^∗^

	**Rutin (mg g^-1^)**			**P-coumaric acid (mg g^-1^)**		
		
CK	1.30 ± 0.06	1.15 ± 0.05	1.25 ± 0.06	0.16 ± 0.01	0.20 ± 0.01	0.24 ± 0.01
O_3_	1.33 ± 0.06	1.34 ± 0.06	1.48 ± 0.07	0.17 ± 0.01	0.21 ± 0.01	0.25 ± 0.01
Percentage change	+2.31	+16.52^∗^	+18.40^∗^	+1.78	+4.25	+7.56
UV-B	1.40 ± 0.07	1.60 ± 0.08	1.70 ± 0.08	0.17 ± 0.01	0.22 ± 0.01	0.26 ± 0.01
Percentage change	+7.69^∗^	+39.13^∗^	+36.00^∗^	+3.79	+8.33	+11.44
O_3_ + UV-B	1.35 ± 0.06	1.53 ± 0.07	1.65 ± 0.08	0.16 ± 0.01	0.21 ± 0.01	0.26 ± 0.01
Percentage change	+3.85	+33.04^∗^	+32.00^∗^	-0.34	+3.35	+10.24


*Plus and minus symbols indicate a positive and negative effects of elevated O_*3*_ and UV-B radiation. Asterisks indicate significant differences for the treatments of elevated O_*3*_, enhanced UV-B and O_*3*_ + UV-B from the control treatment, ^∗^*P* < 0.05.*

Elevated O_3_, UV-B radiation and O_3_ + UV-B showed significant positive effects on ABA concentration during the whole period of soybean growth (**Table 2**). ZR concentration was significantly increased exposed to elevated O_3_, enhanced UV-B radiation and O_3_ + UV-B at stages of branching and podding. ZR concentration was significantly decreased exposed to enhanced UV-B radiation and O_3_ + UV-B at flowering stage. Elevated O_3_, enhanced UV-B radiation and O_3_ + UV-B showed significant negative effects on IAA concentration at podding stage, and on ZR/ABA ratio at flowering stage, and on ratios of IAA/ABA and (IAA+ZR)/ABA at stages of branching, flowering and podding.

**Table 2:** Differences in endogenous hormones of soybean roots in response to control treatment (CK), elevated O_3_ treatment (O_3_), enhanced UV-B treatment (UV-B) and O_3_ + UV-B treatment (O_3_ + UV-B) at stages of branching, flowering, and podding (mean ± SD, *n* = 3).

Treatments	Branching	Flowering	Podding	Branching	Flowering	Podding
	**ABA (μg g^-1^)**			**ZR/ABA**		
		
CK	200.11 ± 8.61	250.68 ± 10.79	340.87 ± 14.70	0.15 ± 0.01	0.14 ± 0.01	0.09 ± 0.01
O_3_	267.36 ± 11.51	315.26 ± 13.57	391.46 ± 16.83	0.13 ± 0.01	0.12 ± 0.01	0.09 ± 0.01
Percentage change	+33.61^∗^	+25.76^∗^	+14.84^∗^	-8.93	-14.20^∗^	-1.04
UV-B	254.96 ± 11.03	294.26 ± 12.73	379.93 ± 16.29	0.12 ± 0.01	0.11 ± 0.01	0.09 ± 0.01
Percentage change	+27.41^∗^	+17.38^∗^	+11.46^∗^	-14.10^∗^	-18.67^∗^	4.39
O_3_ + UV-B	248.15 ± 11.66	322.70 ± 13.94	408.82 ± 17.60	0.14 ± 0.01	0.10 ± 0.01	0.09 ± 0.01
Percentage change	+24.01^∗^	+28.73^∗^	+19.93^∗^	-2.15	-27.77 ^∗^	6.72

	**ZR (ng g^-1^)**			**IAA/ABA**		
		
CK	29.06 ± 1.34	35.28 ± 1.62	29.54 ± 1.36	0.89 ± 0.07	0.52 ± 0.05	0.34 ± 0.03
O_3_	35.36 ± 1.63	38.07 ± 1.75	33.57 ± 1.54	0.46 ± 0.04	0.31 ± 0.03	0.19 ± 0.02
Percentage change	+21.68^∗^	+7.91^∗^	+13.64^∗^	-48.52^∗^	-40.33^∗^	-43.39^∗^
UV-B	31.81 ± 1.46	33.68 ± 1.55	34.37 ± 1.58	0.67 ± 0.06	0.41 ± 0.04	0.22 ± 0.01
Percentage change	+9.44^∗^	-4.53^∗^	+16.35^∗^	-25.09^∗^	-21.93^∗^	-34.01^∗^
O_3_ + UV-B	35.26 ± 1.62	32.81 ± 1.51	37.80 ± 1.74	0.62 ± 0.05	0.39 ± 0.04	0.22 ± 0.02
Percentage change	+21.35^∗^	-7.01^∗^	+27.99^∗^	-30.38^∗^	-24.26^∗^	-35.50^∗^

	**IAA (ng g^-1^)**			**(IAA+ZR)/ABA**		
		
CK	178.87 ± 7.51	130.33 ± 5.47	115.34 ± 4.84	1.04 ± 0.09	0.66 ± 0.06	0.43 ± 0.04
O_3_	123.02 ± 5.17	97.80 ± 4.11	74.99 ± 3.15	0.59 ± 0.05	0.43 ± 0.04	0.28 ± 0.02
Percentage change	-31.22^∗^	-24.96^∗^	-34.99^∗^	-42.99^∗^	-34.77^∗^	-34.76^∗^
UV-B	170.73 ± 7.17	119.44 ± 5.02	84.84 ± 3.56	0.79 ± 0.07	0.52 ± 0.05	0.31 ± 0.02
Percentage change	-4.55	-8.35	-26.45^∗^	-23.55^∗^	-21.23^∗^	-26.18^∗^
O_3_ + UV-B	154.42 ± 6.49	127.06 ± 5.34	89.22 ± 3.75	0.76 ± 0.06	0.50 ± 0.04	0.31 ± 0.02
Percentage change	-13.67	-2.51	-22.64^∗^	-26.44^∗^	-25.01^∗^	-26.89^∗^


*Plus and minus symbols indicate a positive and negative effects of elevated O_*3*_ and UV-B radiation. Asterisks indicate significant differences for the treatments of elevated O_*3*_, enhanced UV-B and O_*3*_ + UV-B from the control treatment, ^∗^*P* < 0.05. ABA, abscisic acid; ZR, zeatin riboside; IAA, 3-indoleacetic acid.*

Elevated O_3_, enhanced UV-B radiation and O_3_ + UV-B significantly inhibited SOD activity at flowering stage, and POD activity at podding stage, and APX activity during the whole period of soybean growth, and CAT activity at podding stage (**Table 3**). Concerning PAL activity, elevated O_3_ showed significant negative effects, and enhanced UV-B radiation showed significant positive effects during the whole period of soybean growth. Elevated O_3_, enhanced UV-B radiation and O_3_ + UV-B showed significant positive effects on LOX activity during the whole period of soybean growth.

**Table 3:** Differences in enzyme activities of soybean roots in response to control treatment (CK), elevated O_3_ treatment (O_3_), enhanced UV-B radiation treatment (UV-B) and O_3_ + UV-B treatment (O_3_ + UV-B) at stages of branching, flowering, and podding (mean ± SD, *n* = 3).

Treatments	Branching	Flowering	Podding	Branching	Flowering	Podding
	**SOD (U g^-1^ h^-1^ FW)**			**CAT (U g^-1^ h^-1^ FW)**		
		
CK	129.62 ± 15.23	103.92 ± 7.57	73.16 ± 7.57	5.65 ± 0.53	4.83 ± 0.28	6.75 ± 0.12
O_3_	97.68 ± 14.89	86.38 ± 6.10	64.30 ± 14.80	3.40 ± 0.09	4.81 ± 0.41	3.75 ± 0.25
Percentage change	-24.64^∗^	-16.88^∗^	-12.1	-39.85^∗^	-0.43	-44.44^∗^
UV-B	128.91 ± 19.24	90.59 ± 5.03	71.00 ± 2.44	5.35 ± 0.38	4.33 ± 0.03	4.75 ± 0.12
Percentage change	-0.55	-12.83^∗^	-2.94	-5.17	-10.34	-29.63^∗^
O_3_ + UV-B	77.10 ± 15.13	81.76 ± 12.69	68.01 ± 5.43	2.94 ± 0.03	4.77 ± 0.40	3.50 ± 0.43
Percentage change	-40.52^∗^	-21.32^∗^	-7.03	-47.97^∗^	-1.29	-48.15^∗^

	**POD (U g^-1^ h^-1^ FW)**			**PAL (U g^-1^ h^-1^ FW)**		
		
CK	65.87 ± 10.07	187.80 ± 12.88	348.33 ± 15.45	1.73 ± 0.43	2.82 ± 0.10	4.02 ± 0.05
O_3_	54.20 ± 9.49	159.47 ± 22.72	233.00 ± 22.52	1.26 ± 0.10	2.17 ± 0.03	3.30 ± 0.11
Percentage change	-17.71^∗^	-15.09^∗^	-33.11^∗^	-27.24^∗^	-23.18^∗^	-17.87^∗^
UV-B	63.80 ± 15.60	163.73 ± 14.14	295.00 ± 9.18	2.14 ± 0.23	3.32 ± 0.05	4.79 ± 0.02
Percentage change	-3.14	-12.82	-15.31^∗^	+23.72^∗^	+17.53^∗^	+19.04 ^∗^
O_3_ + UV-B	48.47 ± 10.82	143.40 ± 11.29	221.13 ± 9.78	1.90 ± 0.14	2.94 ± 0.03	4.20 ± 0.04
Percentage change	-26.42^∗^	-23.64^∗^	-36.52^∗^	9.7	4.26	4.53

	**APX (U g^-1^ h^-1^ FW)**			**LOX (OD g^-1^ min^-1^ FW)**		
		
CK	2.06 ± 0.11	1.89 ± 0.12	1.07 ± 0.05	2.06 ± 0.08	4.51 ± 0.18	6.28 ± 0.26
O_3_	1.39 ± 0.17	1.30 ± 0.15	0.43 ± 0.01	3.86 ± 0.16	5.10 ± 0.21	7.46 ± 0.31
Percentage change	-32.72 ^∗^	-31.38^∗^	-59.93^∗^	+87.38^∗^	+13.03^∗^	+18.76^∗^
UV-B	1.64 ± 0.21	1.47 ± 0.22	0.87 ± 0.01	4.98 ± 0.20	8.06 ± 0.33	11.56 ± 0.47
Percentage change	-20.40^∗^	-22.20^∗^	-18.24^∗^	+141.75^∗^	+78.63^∗^	+83.99^∗^
O_3_ + UV-B	1.37 ± 0.13	1.19 ± 0.24	0.25 ± 0.01	4.08 ± 0.17	6.95 ± 0.28	9.55 ± 0.39
Percentage change	-33.39^∗^	-37.25^∗^	-76.87^∗^	+98.06^∗^	+54.03^∗^	+52.00^∗^


*Plus and minus symbols indicate a positive and negative effects of elevated O_*3*_ and/or UV-B radiation. Asterisks indicate significant differences for the treatments of elevated O_*3*_, enhanced UV-B and O_*3*_ + UV-B from the control treatment, ^∗^*P* < 0.05. SOD, superoxide dismutase; POD, peroxidase; APX, ascorbate peroxidase; CAT, catalase; PAL, phenyl alanine ammonia; LOX, lipoxygenase.*

**FIGURE 4:**
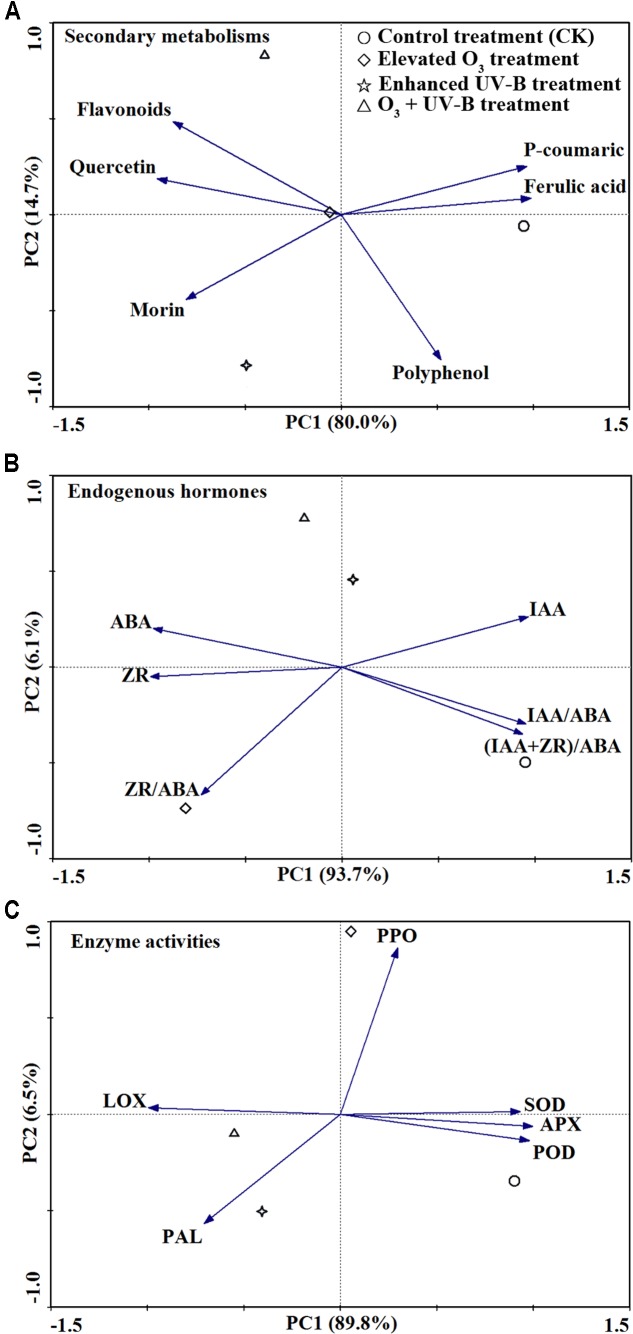
Principal component analysis analysis of secondary metabolisms **(A)**, endogenous hormones **(B)** and enzyme activities **(C)** in response to control treatment (CK), elevated O_3_ treatment, enhanced UV-B radiation treatment and O_3_ + UV-B treatment at stages of branching, flowering, and podding.

According to PCA analysis, the effects of elevated O_3_, UV-B radiation and O_3_ + UV-B on the concentrations of secondary metabolisms, endogenous hormones and enzyme activities were differed from each other (**Figure 4**). In the PCA analysis of all secondary metabolisms, CK treatment and elevated O_3_ treatment were separated on the first axis, and the UV-B radiation treatment and O_3_ + UV-B treatment were separated on the second axis. As to endogenous hormones, the CK treatment was separated on the first axis, and the elevated O_3_ treatment, UV-B radiation treatment and O_3_ + UV-B treatment were separated on the second axis. Furthermore, the first axis discriminated CK treatment and O_3_ + UV-B treatment, and the elevated O_3_ treatment and UV-B radiation treatment were separated on the second axis in the PCA analysis of all enzyme activities.

Multivariate analysis of secondary metabolisms and endogenous hormones showed a significant interactive effect of elevated O_3_ and enhanced UV-B on all parameters (O_3_ × UV-B term all at *P* < 0.05; **Table 4**). There were significant interactive effects of elevated O_3_ concentration and enhanced UV-B radiation on the activities of APX and LOX (O_3_ × UV-B term at *P* = 0.044, *P* < 0.001, respectively).

**Table 4:** *P*-values of 3-way ANOVA results showing the effect of the elevated O_3_ concentration, the enhanced UV-B radiation and the growth stage on secondary metabolisms, endogenous hormones, and enzyme activities of soybean roots.

	O_3_	UV-B	Stage	O_3_ × UV-B	O_3_ × Stage	UV-B × Stage	O_3_ × UV-B × Stage
**Secondary metabolisms**							
Total flavonoids	0.005	<0.001	0.040	0.002	0.101	0.442	0.081
Total polyphenol	0.647	0.06	<0.001	<0.001	0.949	0.372	0.041
Quercetin	0.001	<0.001	<0.001	<0.001	0.094	0.381	0.371
Ferulic acid	0.64	0.006	<0.001	0.006	0.736	0.207	0.719
Rutin	0.048	<0.001	<0.001	<0.001	0.197	<0.001	0.156
P-coumaric acid	0.663	0.03	<0.001	0.034	0.624	0.259	0.943
**Endogenous hormones**							
ABA	<0.001	<0.001	<0.001	<0.001	0.312	0.903	0.005
ZR	<0.001	0.09	0.009	0.021	0.009	<0.001	0.608
IAA	<0.001	0.025	<0.001	<0.001	<0.001	<0.001	0.808
ZR/ABA	<0.001	<0.001	<0.001	<0.001	<0.001	<0.001	<0.001
IAA/ABA	<0.001	<0.001	<0.001	<0.001	<0.001	<0.001	<0.001
(IAA+ZR)/ABA	<0.001	<0.001	<0.001	<0.001	<0.001	<0.001	<0.001
**Enzyme activities**							
SOD	<0.001	0.142	<0.001	0.825	0.02	0.362	0.252
CAT	<0.001	0.057	0.243	0.432	0.001	0.617	0.492
POD	<0.001	0.033	<0.001	0.775	0.23	0.94	0.696
PAL	<0.001	<0.001	<0.001	0.054	0.096	0.074	0.852
APX	<0.001	0.001	<0.001	0.044	0.352	0.854	0.361
LOX	<0.001	<0.001	<0.001	<0.001	<0.001	0.482	<0.001


*O_*3*_: elevated O_*3*_ treatment; UV-B: enhanced UV-B radiation; Stage: growth stage. ABA, abscisic acid; ZR, zeatin riboside; IAA, 3-indoleacetic acid; SOD, superoxide dismutase; POD, peroxidase; APX, ascorbate peroxidase; CAT, catalase; PAL, phenyl alanine ammonia; LOX, lipoxygenase.*

### Modulation of Biochemical Properties on Root Growth and Activity

Redundancy analyses analyses showed that the explained variation in secondary metabolisms and enzyme activities by effects of elevated O_3_ and UV-B radiation on root biomass was significant (*P* = 0.024, *P* = 0.002, *P* = 0.044, *P* = 0.026, respectively; **Table 5**). The explained variation in secondary metabolisms and endogenous hormones by effects of O_3_ + UV-B on root biomass was significant (all at *P* = 0.002). Concerning nodule biomass, explained variations in secondary metabolisms by UV-B radiation effects and in endogenous hormones by O_3_ + UV-B effect were significant (*P* = 0.038 and *P* = 0.002, respectively).

**Table 5:** Results of redundancy analysis (RDA) of the modulation of secondary metabolisms, endogenous hormones and enzyme activities in response to elevated O_3_ treatment (O_3_), enhanced UV-B radiation treatment (UV-B) and O_3_ + UV-B treatment (O_3_ + UV-B) on root biomass, root nodule biomass and root activities.

		Root biomass			Root nodule biomass			Root activities		
				
Treatments	Index	*F*	*P*	Explained (%)	*F*	*P*	Explained (%)	*F*	*P*	Explained (%)
O_3_	Secondary metabolisms	2.58	**0.024**	**33.00**	1.69	0.202	19.00	0.66	0.372	9.40
	Endogenous hormones	0.99	0.464	15.80	3.25	0.140	19.60	0.80	0.290	4.40
	Enzyme activities	5.28	**0.002**	**3.60**	0.71	0.418	6.30	3.57	**0.082**	**18.30**
UV-B	Secondary metabolisms	8.30	**0.044**	**34.20**	8.33	**0.038**	**19.70**	0.32	0.752	9.70
	Endogenous hormones	1.41	0.168	11.90	1.47	0.218	19.00	1.84	0.220	4.80
	Enzyme activities	4.50	**0.026**	**3.40**	0.49	0.388	5.50	0.50	0.292	17.20
O_3_ + UV-B	Secondary metabolisms	5.40	**0.002**	**33.60**	4.11	0.132	20.40	1.20	0.334	9.20
	Endogenous hormones	2.94	**0.002**	**12.80**	4.54	**0.002**	**18.70**	0.49	0.504	4.60
	Enzyme activities	1.93	0.132	3.20	2.52	0.112	6.50	2.01	0.252	18.00


*Explained variance is based on the sum of all canonical eigen values. *P*-values are based on a Monte-Carlo permutation test with 999 permutations, and restricted for split-plot design. Significant differences were labeled with bold.*

## Discussion

Our study found that elevated O_3_ concentration and enhanced UV-B radiation individually decreased root biomass at each developmental stages (branching stage, flowering stage, and podding stage). Root growth is highly dependent on carbohydrate availability (Muller et al., 1998; Walter et al., 2009). The balance between carbohydrate assimilation, storage, and growth and the allocation between shoot and root are crucial for root growth (Schurr et al., 2006). Elevated O_3_ is known to produce a decline in the availability of carbon in leaves by impairing stomatal function and influencing Rubisco carboxylation capacity, which can alter carbon balance of plants, decreasing the allocation of carbon to roots and shoots and leading to an altered biomass of shoots and roots (Andersen, 2003; Fiscus et al., 2005; Matyssek et al., 2008). Meanwhile, enhanced UV radiation also could alter biomass of shoots and roots in many plant species, because exceeding ambient UV radiation intensity could directly affect photosynthesis systems and phyto-hormones and indirectly impact stomatal closure (Kakani et al., 2003; Yadav et al., 2017). Thus, to better understanding the mechanism of root growth in response to elevated O_3_ and enhanced UV-B radiation, plant biomass allocation (root biomass: shoot biomass and root nodule biomass: shoot biomass) needs to be further studied. Furthermore, the significant effects of O_3_ × UV-B were shown in the present study indicating that the combined effects of elevated O_3_ concentration and enhanced UV-B radiation were synergistic on root biomass of soybean. Thus, combined effects of elevated O_3_ and enhanced UV-B significantly exacerbated the decreased biomass of soybean roots.

Moreover, in accordance with our hypothesis, biomass of root nodule significant decreased in the presence of both treatments of elevated O_3_ and enhanced UV-B radiation individually or in combination. Decreased biomass of root nodule might decrease N uptake of roots, and therefore might exacerbate root senescence or death. Meanwhile, the effects of O_3_ × UV-B on nodule biomass were insignificant in the present study indicating that the combined effects of elevated O_3_ and enhanced UV-B on nodule biomass were additive.

Root activity is a physiological index reflecting the ability of roots to absorb water and nutrients, to synthesize certain compounds, and to either oxidize or reduce elements in the surrounding rhizosphere. Previous studies showed that enhanced UV-B radiation and elevated O_3_ significantly decreased root activity of rice (Dai et al., 1992). Similarly, in present study, elevated O_3_ and enhanced UV-B radiation individually as well as in combination inhibited the root activity at each developmental stages (branching stage, flowering stage, and podding stage). Meanwhile, the inhibition effect of coupling elevated O_3_ and enhanced UV-B radiation on root activity was more significant (55.7%) than individual treatment (11.2 and 39.9%, respectively). However, the effects of O_3_ × UV-B on root activity were insignificant in the present study indicating that the combined effects of elevated O_3_ and enhanced UV-B on root activity were simply additive. Moreover, elevated O_3_ and combination effects of O_3_ and UV-B radiation significantly decreased POD concentration of soybean roots during the whole incubation in present study. Previous studies showed that hydrogen peroxide (H_2_O_2_) could be oxidized by α-naphthylamine in the presence of iron oxidase (mainly POD) (Schopfer and Liszkay, 2006), thus, there was a positive correlation between POD activity and oxidation intensity of α-naphthylamine.

O_3_ is known to affect the plasma function by disorganizing the membrane structure and altering membrane permeability through lipid peroxidation and electrolyte leakage (Calatayud et al., 2003). In the present study, MDA concentration and relative electrical conductivity of soybean roots were drastically enhanced by elevated O_3_ at three developmental stages, indicating that O_3_ intensified accumulation of ROS (reactive oxygen species) induced by oxidative stress and degree of lipid peroxidation of root issue membrane throughout the whole growing period. Similarly, MDA concentrations and relative electrical conductivity of soybean roots during the whole period of soybean growth were also enhanced by enhanced UV-B, indicating that enhanced UV-B radiation also induced oxidative stress. Furthermore, O_3_ + UV-B radiation generally displayed higher MDA concentration and relative electrical conductivity than enhanced UV-B radiation during the whole growing period of soybean roots, and according to multivariate analysis, the effects of O_3_ × UV-B on MDA concentration and relative electrical conductivity were significant in the present study. Thus supplemental O_3_ aggravated the oxidative effects of UV-B radiation.

Phenols are mainly responsible for providing plant pest and disease resistance and protection. In present study, there generally was no significant change in phenols concentration at three developmental stages. Differently, Ambasht and Agrawal (2003) and Tripathi et al. (2011) reported increases in phenols of crop leaves under treatment of UV-B radiation and/or O_3_. Moreover, the present study demonstrated a considerable difference in the flavonoids concentration in response to elevated O_3_ and/or enhanced UV-B radiation. Li B. et al. (2012) also found that enhanced UV-B radiation increased flavonoids concentration of roots. Some studies found that flavonoids (such as kaempferol) were an inhibitor of auxin transport (Peer et al., 2004; Taylor and Grotewold, 2005), and auxin was a key player on organizing node for environmental/hormonal modulation of root hair growth (Lee and Cho, 2013). In the present study, three treatments of elevated O_3_, enhanced UV-B radiation and elevated O_3_ + enhanced UV-B radiation significantly increased IAA concentration at podding stage. The accumulation of flavonoids presumably resulted in the reduction of roots development through inhibiting auxin transport in the root (Brown et al., 2001). Quercetin and rutin (sometime called vitamin P) was known to have strong antioxidant activity which could alleviate the damage exposed to UV-B radiation (Edreva, 2005). In the present study, concentrations of quercetin and rutin of roots were significantly increased at stages of flowering and podding. Similarly, Huang et al. (2016) showed that contents of quercetin and rutin of fine roots were increased when exposed to enhanced UV-B radiation.

Plants have several enzymatic antioxidants (such as SOD, CAT, POD, and APX) to defend themselves against oxidative stress (Gill and Tuteja, 2010). Previous studies have found various trends in enzyme activity of leaves in response to elevated O_3_ concentration and/or enhanced UV-B radiation mainly based on the above-ground system. Kumari et al. (2009) showed that a reduction in SOD activity of leaves of *Abelmoschus esculentus* L. after 10 days and then subsequent increment after 20 days exposed to enhanced UV-B radiation (10.4 kJ m^-2^). Rai and Agrawal (2008) found a significant increase in SOD activity in rice leaves under elevated O_3_. Tripathi et al. (2011) found an increment in activity of SOD, CAT, and APX of leaves of *Linum usitatissimum* L. in response to O_3_ (+10 ppb) and UV-B radiation (+7.2 kJ m^-2^ d^-1^) individually as well as in combination. Rao et al. (1996) and Baumbusch et al. (1998) also reported increased CAT and POD activity in UV-B radiation, O_3_, and UV-B + O_3_ in leaves, while Rao et al. (1996) did not find any major change in CAT activity after O_3_ treatment (200 ppb). In the present study, activities of SOD, CAT, POD, and APX of roots were all significantly decreased in response to O_3_ and UV-B radiation individually as well as in combination, while, there were insignificant effects of O_3_ × UV-B on the activity of SOD, CAT, and POD. Thus, the combined effects of elevated O_3_ and enhanced UV-B on the activity of SOD, CAT and POD were simply additive. SOD is the primary enzyme responsible for the dismutation of superoxide anions and hence decreasing the risk of hydroxyl radicals from superoxide anions (Arora et al., 2002). And for detoxification of H_2_O_2_, other enzymes (such as POD, APX, and CAT) were found inside the cell. Production rate of superoxide anions and hydroxyl radicals increased significantly with the high dose of enhanced UV and/or elevated O_3_, which might result in enzyme activity exceeding its threshold. Thus, activities of SOD, CAT, POD, and APX were all significantly decreased in the present study.

According to redundancy analysis, secondary metabolisms showed stronger relationship with root growth in response to elevated O_3_, enhanced UV-B radiation and elevated O_3_ + enhanced UV-B radiation compared to hormones and enzyme activity, probably because the secondary metabolites are more sensitive to elevated O_3_ and enhanced UV-B radiation compared to hormones and enzyme activity, especially for annual legumes. Secondary metabolites (such as flavonoids, phenol) are considered as a first line to limit the defense of environmental change (Bartwal et al., 2013). UV-B radiation and O_3_ lead to not only ROS generation and oxidative stress, but also changes of hormones and activity of antioxidant enzyme. However, previous studies showed that the exact set of enzymes activated is dependent on the plant species and no consensus has been reached as to the most important antioxidant enzyme system in defense of environmental changed (Soheila, 2000; Caldwell et al., 2007). Thus, for annual legumes in present study, the variation of root growth exposed to O_3_ and/or UV-B radiation was mostly associated with flavonoids.

In the present study, the effects of O_3_ × UV-B on root biomass, flavonoids, and hormones were significant indicating that the changes of root biomass, flavonoids, and hormones in response to coupling UV radiation and O_3_ were always greater than that in response to individual effects. Thus, supplemental elevated O_3_ might exacerbate the damage of enhanced UV-B on soybean root growth, contrary to some previous studies mainly based on the above-ground system (Zeuthen et al., 1997; Baumbusch et al., 1998; Tripathi et al., 2011). For example, Zeuthen et al. (1997) found that UV-B and O_3_ in combination enhanced the negative effects on photosynthesis of beech compared with UV-B and O_3_ alone. Baumbusch et al. (1998) reported that, generation of ROS of pine leaf was counteracted by combination effects. Tripathi et al. (2011) showed that generation of ROS of linseed in response to combined effects of UV-B and O_3_ was lower than that to individual effects.

## Conclusion

The major objective of our study was to assess the effects of elevated O_3_ and enhanced UV-B radiation individually as well as in combination on root growth and activity of soybean, and we found that elevated O_3_ and enhanced UV-B radiation individually inhibited root growth and activity of soybean. Elevated O_3_ and enhanced UV-B individually as well as in combination induced oxidative damage of soybean roots leading to changes in secondary metabolites (especially flavonoids), endogenous hormone and enzyme activities of soybean roots. We found that, compared to endogenous hormones and enzyme activity, secondary metabolisms (especially flavonoids) showed stronger relationship with root growth in response to elevated O_3_, enhanced UV-B radiation and their combination. Consequently, the inhibited root growth was mostly associated with flavonoids. Moreover, the effect of UV-B + O_3_ was generally greater than those of their individual stresses on root biomass, flavonoids, and hormones.

## Author Contributions

BM and R-RT detected the changes of secondary metabolites of soybean roots. YW detected the changes of endogenous hormone of soybean roots. WW and J-SY completed the experiment of the effect of elevated ozone and enhanced UV-B on root growth of soybean. T-HZ guided the whole research as the corresponding author.

## Conflict of Interest Statement

The authors declare that the research was conducted in the absence of any commercial or financial relationships that could be construed as a potential conflict of interest.

## Abbreviations

ABA: abscisic acid
APX: ascorbate peroxidase
CAT: catalase
IAA: 3-indoleacetic acid
LOX: lipoxygenase
MDA: malondialdehyde
PAL: phenylalanine ammonia
POD: peroxidase
SOD: superoxide dismutase
ZR: zeatin riboside
